# Psychological costs of inadequate cervical smear test results

**DOI:** 10.1038/sj.bjc.6602224

**Published:** 2004-11-09

**Authors:** D P French, E Maissi, T M Marteau

**Affiliations:** 1 Department of Psychology (at Guy's), Institute of Psychiatry, Kings College London, UK

**Keywords:** cervical smear test result, inadequate, state anxiety, satisfaction with information, attendance

## Abstract

The purpose of the study is to investigate, for the first time, the psychological impact of an inadequate smear test result. A prospective questionnaire design was used, and the setting was a single English cervical screening laboratory. Two groups of women receiving either a normal test result (*n*=226) or either a first or nonconsecutive inadequate smear test result (*n*=180) participated. The main outcome measures included State anxiety (STAI) and concern about test result, assessed within 4 weeks of receipt of results, and attendance for a repeat cervical smear within 3 months. Compared to women with normal test results, women with inadequate smear test results had higher state anxiety (*P*=0.025), were more concerned about their results (*P*<0.001), perceived themselves to be at higher risk of cervical cancer (*P*=0.016), and felt less satisfied with the information they had received about their test results (*P*<0.001). The only predictor of attendance for a repeat smear test following an inadequate smear test result was state anxiety (*P*=0.011): nonattenders had higher levels of state anxiety in response to their initial test results. In conclusion, in this first study to assess the psychological impact of receiving an inadequate smear test result, we have shown that it raises state anxiety and concern to levels similar to those found in women with abnormal smear test results. Of particular concern is that anxious women are less likely to attend for a repeat smear test within the recommended time frame. Given the millions of women each year receiving this test result, research is now needed to ascertain how the anxiety associated with this result can be avoided.

World wide, millions of women each year undergoing a cervical smear test receive a test result indicating that the test was inadequate or unsatisfactory. In the UK's National Health Service Cervical Screening Programme in 2002–2003, the overall percentage of inadequate smears was 9.4%, resulting in over 300 000 women receiving an inadequate test result (Department of Health, 2003). A smear test may be reported as inadequate for a number of reasons, the most common being masking of epithelial cell detail by pus or insufficient epithelial cells being present for accurate assessment. A first or nonconsecutive inadequate smear test result is a relatively ambiguous piece of information, whose true prognostic and clinical significance is currently not fully understood (Hock *et al*, 2003). Current policy in the UK is for smear tests reported as inadequate to be repeated as soon as possible and that after three consecutive inadequate smears women should be referred for a colposcopic examination of the cervix.

While it is well documented that following the receipt of abnormal smear test results, or referral for colposcopy, women experience high levels of anxiety (Marteau *et al*, 1990; Lerman *et al*, 1991; Rogstad, 2002), we have been unable to identify any published reports of the psychological impact of receiving an inadequate smear test result. Given the high incidence of inadequate smear test results, a description of the psychological costs of this test result is essential in evaluating the cervical screening programme. These costs may be behavioural as well as emotional: for example, 15% of women who were recalled for further examination after routine mammography did not attend their next routine breast-screening appointment, compared with 8% of women who received a clear result (Brett and Austoker, 2001). There is also reason to believe that emotional and behavioural effects of receiving an inadequate smear test result may be related: previous research has found that the most important predictor of nonattendance for cervical screening is fear of the consequences of the investigation (Murray and McMillan, 1993).

The aims of the current study are (a) to assess whether receipt of an inadequate smear test result raises general levels of anxiety and test- result-specific concern, (b) to examine possible predictors of these, and (c) to assess whether the emotional effects of an inadequate smear test result affect attendance for a repeat test. Examining the predictors of distress and nonattendance is a first step towards identifying how such tests results can be presented to avoid adverse psychological and clinical outcomes. Specifically, we hypothesised that within a few weeks of receiving their smear test results, women with normal results would have anxiety and concern scores in the normal range (anxiety score=35; concern score=5), while women with inadequate smear test results would have raised anxiety and concern scores (anxiety score=38; concern score=11), similar to those found in women with borderline or mildly dyskaryotic smear test results (Maissi *et al*, 2004).

## METHODS

### Design

All women receiving an inadequate smear test result from a single laboratory over a 7-week period were invited to participate, along with a comparison group, selected from all women who received a normal smear test result from the same laboratory over the same period. The laboratory used conventional cytology.

### Participants

The final sample comprised 180 women who had received either their first or a nonconsecutive inadequate smear test result, and 226 women who had received a normal smear test result. Given a population standard deviation (s.d.) of 12, 200 women per group were required to detect the hypothesised effects (*d*=0.25), with 80% power at the 5% level of significance. From similar previous research, we assumed there would be a 60% response rate, requiring 336 women to be invited to participate in each group.

The women in the study had a mean age of 40.4 years (s.d.=12.8), with the majority (94.5%) being white. They had a range of education, with 80 women (19.7%) having no qualifications, 161 (39.7%) having GCSE or GCE (O or A levels) qualifications, and 163 (40.1%) having at least some higher education. For 37 women (9.1%), this was their first cervical smear, while for 366 it was not. Women who received an inadequate smear test result did not significantly differ from those who received a normal smear test result in any of the demographic variables assessed.

### Procedure

All women with an inadequate smear test result identified by the cytology screening laboratory office of Sheffield Family Health Services over a 7-week period were invited to participate in the study. The comparison sample comprised women who received a normal smear test result from the same laboratory over the same period. Over this period, the population of all women who received a normal smear test result was entered into the Sheffield Family Health Services database. A weighted sample of women was drawn from this population, stratified by postcode. As selection was performed by postcode instead of name, anonymity of participants was ensured. An invitation letter and a detailed study information sheet were posted to women with their smear test results, sent by the cytology screening laboratory. Women not wishing to participate were allowed 2 days to decide and post an opt out slip back to the screening laboratory office. Up to two reminders were sent after 7 and 14 days to women who had not returned their questionnaires. Data on attendance for a repeat cervical smear test within 3 months of an initial inadequate smear test result were obtained from the records of Sheffield Family Health Services. Ethical approval to conduct this study was granted by the South Sheffield Research Ethics Committee (Ref. no.: 03/008).

### Materials

All women invited for screening were sent a national leaflet explaining the purpose of screening and possible results, including information about inadequate smear test results.

#### Normal smear test results letter

All women with this test result received a letter with the following explanation:

‘The result of your recent cervical smear test was normal. If you are aged between 20 and 64 years, you will be invited by letter to have another smear test in 3 years time unless your doctor advises an earlier smear. However, the smear test is not infallible and so if you experience any unusual bleeding or symptoms that concern you before your next smear, or if you require any further information regarding cervical smears, please contact your GP or Central Health Clinic’.

#### Inadequate smear test results letter

All women with an inadequate smear test result received a letter with the following explanation:

‘It has not been possible to obtain a result from your recent cervical smear test. This is quite common, for a number of reasons, for example, the smear may not contain enough material for the laboratory to analyse. Your smear taker will be able to explain this in more detail. It is important that you contact the surgery, clinic or hospital where you attended for your smear to arrange for a repeat test at a time that suits you. You should receive your result within 8 weeks. We stress that there is no reason to be worried, but if you require any medical advice please do not hesitate to contact your family doctor (GP) or practice nurse, or the clinic or hospital where the test was performed’.

### Measures

*State anxiety* was assessed using the six-item short-form of the state scale of the Spielberger State-Trait Anxiety Inventory (S-STAI-6), with the obtained score multiplied by 20/6 to give a scale range from 20 to 80 (Marteau and Bekker, 1992) for those women who completed each item of the scale (*n*=354). Five women had completed at least three of the items (50%), hence their scores were prorated increasing the number of analysed cases to 359. The population norm for women is 35, with scores above 49 being found in patients with a diagnosis of anxiety disorder (Spielberger *et al*, 1970). The internal reliability (Cronbach's *α*) of the scale in this sample was 0.84 (*n*=354).

*Concern about the smear result* was assessed using two seven-point rating scales (range 2–14) asking women (a) how concerned, and (b) how reassured they felt about their smear test result. Higher scores indicate more concern (*α*=0.72, *n*=401).

*Perceived stressfulness* about attending for a subsequent (repeat or routine) smear test appointment was assessed using a seven-point scale. Higher scores indicate stronger agreement with the statement ‘Having a smear test again would be stressful for me’.

*Perceived relative risk of developing cervical cancer* was assessed by asking women to rate whether, compared with other women of their age, their risk of developing cervical cancer in the next 10 years was (a) much higher, (b) a bit higher, (c) about the same, (d) a bit lower, or (e) much lower.

*Satisfaction with information about the smear test result* was assessed using four seven-point scales asking women (a) how well informed they felt about their smear test result, (b) how satisfied they were with the amount of information they had been given, (c) how confusing, and (d) how clear they felt that information was. Higher scores on the four-item scale (range 4–28; *α*=0.83, *n*=400) indicate higher satisfaction.

*Understanding of smear test result* was assessed by asking women to select the one among the following eight options which best described what their test result meant for their current cervical health: (a) I definitely do not have cervical cancer, (b) I am very unlikely to have cervical cancer, (c) I am unlikely to have cervical cancer, (d) I am likely to have cervical cancer, and (e) I am very likely to have cervical cancer, (f) I have cervical cancer, (g) This result does not tell me anything about whether or not I have cervical cancer, and (h) I do not know what my smear test result means. The correct response for women receiving a normal smear test result was (b) or (c). For women receiving an inadequate smear test result the correct response was (g), (b), or (c).

*Demographic information:* Age, highest educational achievement, ethnicity, and whether this most recent smear test was their first one or not were assessed.

### Analysis

Differences in demographic and psychological variables between the two test result groups and between attenders and nonattenders for repeat smear tests were assessed using *χ*^2^ tests and independent sample *t*-tests. A nonparametric Mann–Whitney ‘*U*’-test was conducted to assess group differences in perceived comparative risk of developing cervical cancer. Stepwise multiple linear regression was used to identify the best predictors of state anxiety and concern in the inadequate smear test result group only.

## RESULTS

### Response rates

A total of 16 women with normal test results and 11 women with inadequate smear test results opted out of the study. A total of 674 women, 338 with a normal and 336 with an inadequate smear test result, were sent a questionnaire within a week of receiving their test results. A total of 427 women (63%) returned questionnaires: 226 (67%) women with a normal, and 201 (60%) women with an inadequate smear test result. For the majority of women in the inadequate smear test result group (*n*=180, 89.6%), this was either their first inadequate smear test result or one that proceeded at least one normal test result. For 17 (8.5%) women, this was their second consecutive inadequate smear test result, and for four women (2%) it was their third. The final sample of 406 women comprised of 226 women with a normal, and 180 women with a first, or nonconsecutive, inadequate smear test result.

### Outcomes

State anxiety scores for both groups of women were within the normal range. The mean concern scores were low for women receiving a normal test result (4.8, in a possible range of scores of 2–14), but above the mid point for women receiving an inadequate smear test result. As predicted, inadequate smear test results were associated with higher anxiety and concern about the test result (see Table 1

**Table 1:** Emotional outcomes and their predictors (mean (s.d.), % (*n*)) according to smear test result

	**Smear test result groups**			
	**Inadequate (*n*=180)**	**Normal (*n*=226)**	**Test statistics**	***P*-values**
State anxiety: mean (s.d.)	37.8 (13.1)	34.8 (12.4)	*t*=−2.254	**0.025**
Concern: mean (s.d.)	8.7 (2.9)	4.8 (2.5)	*t*=−14.620	**<0.001**
Perceived stressfulness about next smear test: mean (s.d.)	3.5 (2.0)	2.8 (2.0)	*t*=−3.391	**0.001**
Satisfaction with information: Four-item scale: mean (s.d.)	19.2 (5.6)	22.7 (4.5)	*t*=6.989	**<0.001**
Perceived risk of developing cervical cancer: %(*n*)^a^				
(a) Much higher than average	2 (3)	3 (6)		
(b) A bit higher than average	15 (27)	9 (19)		
(c) Same as average	70 (123)	67 (148)	*z*=−2.4111	**0.016**
(d) A bit lower than average	8 (15)	13 (30)		
(e) Much lower than average	5 (8)	8 (17)		

a Mann–Whitney ‘*U*’ test.

*P*-values reaching statistical significance (*P*<0.05) in bold.

). Women with an inadequate smear test result also felt that attending for their next smear test appointment would be more stressful compared with women receiving a normal test result. Both groups, however, had scores below the mid point, suggesting that neither group felt extremely stressed about a subsequent smear test appointment.

The majority of women in both test result groups perceived their risks of developing cervical cancer in the future as average (see Table 1). The two groups differed, however, in their relative risk perceptions, with more women receiving an inadequate smear test result perceiving their risks as higher.

Overall, women in both smear test result groups reported feeling satisfied with the test result information, with mean scores above the scale mid point (see Table 1). However, women receiving an inadequate smear test result reported less satisfaction than women receiving a normal test result.

More than half of the women with normal test results reported correctly that their results meant that they were unlikely to have cervical cancer (*n*=154, 68.4%) (Table 2

**Table 2:** Percentage (*n*) of respondents in the normal and inadequate smear test result groups endorsing statements indicating understandings of the meaning of their latest test result, and their associated anxiety and concern (mean, s.d.)

	**Smear test result groups**					
	**Inadequate (*n*=180)**			**Normal (*n*=226)**		
		**Anxiety**	**Concern**		**Anxiety**	**Concern**
**Understanding of test result**	**% (*n*)**	**Mean (s.d.)**	**Mean (s.d.)**	**% (*n*)**	**Mean (s.d.)**	**Mean (s.d.)**
I definitely do not have cervical cancer	5 (9)	35.9 (16.1)	6.0 (2.7)	23 (52)	37.1 (11.6)	4.4 (2.8)
I am very unlikely to have cervical cancer	9 (16)	40.0 (10.4)	5.9 (3.2)	42 (95)	33.1 (11.8)	4.0 (2.0)
I am unlikely to have cervical cancer	7 (13)	36.7 (9.6)	7.3 (2.1)	26 (59)	34.3 (13.1)	5.8 (2.4)
I am likely to have cervical cancer	1 (1)	43.3	12.0	0	0	0
I am very likely to have cervical cancer	0	0	0	0	0	0
I have cervical cancer	0	0	0	0	0	0
This result does not tell me anything about whether or not I have cervical cancer	72 (125)	37.5 (13.3)	9.3 (2.6)	4 (8)	32.2 (10.9)	5.4 (3.1)
I do not know what my result means	6 (11)	45.2 (14.9)	10.2 (1.9)	5 (11)	42.2 (16.8)	6.7 (3.0)
Missing	3 (5)	28.3 (9.6)	6.5 (3.1)	0 (1)	40	—

). However, a significant minority (*n*=52, 23.1%) erroneously believed that their results meant that they definitely did not have cervical cancer. In the inadequate smear test result group, 71% (*n*=125) of women correctly reported their test results as uninformative about their current cervical health. Levels of concern were particularly high in these women. Perceived risk of developing cervical cancer was also higher in women who reported their results as uninformative compared with those who reported them as informative. A small percentage of women in both groups (4.9% in the normal test result group, 6.3% in the inadequate smear test result group) reported not knowing what their test results meant. These women experienced the highest levels of anxiety and concern.

Two stepwise multiple linear regressions were conducted to identify the predictors of state anxiety (Table 3

**Table 3:** Stepwise multiple linear regression for state anxiety in the inadequate smear test result group (*n*=153)

**Step**	**Variables**	**Partial correlation**	**Initial *β***	**Final *β***	**Increased *R*^2^**	**Unadjusted *R*^2^**
1	a. Age	−0.044	−0.155	−0.052		
	b. Education	0.029	0.077	0.03		
	c. Smear history	0.094	0.072	0.108	0.021	0.021
2	d. Perceived risk	−0.153	−0.165*	−0.154*	0.027	0.048
3	e. Understanding	−0.13	−0.075	−0.135	0.005	0.054
4	f. Satisfaction	−0.367		−0.392***	0.135	0.189

*Education*: Higher education and degree (1) *vs* all other qualifications (including no qualifications) (2).

*Smear history*: Target smear was first (1) *vs* not the first/don't know (2).

*Understanding*: Response (g) coded as 2; all other responses coded as 1.

*Final adjusted* *R*^2^=0.155;

* *P*<0.05;

*** *P*<0.001.

) and concern (Table 4

**Table 4:** Stepwise multiple linear regression for concern about test result in the inadequate smear test result group (*n*=169)

**Step**	**Variables**	**Partial correlation**	**Initial *β***	**Final *β***	**Increased *R*^2^**	**Unadjusted *R*^2^**
1	a. Age	−0.074	−0.244*	−0.087		
	b. Education	−0.093	−0.084	−0.097		
	c. Smear history	0.013	−0.013	0.015	0.078	0.078
2	d. Perceived risk	−0.026	−0.066	−0.026	0.004	0.083
3	e. Understanding	0.164	0.262**	0.171*	0.065	0.148
4	f. Satisfaction	−0.457		−0.493***	0.209	0.357

*Education*: Higher education and degree (1) *vs* All other qualifications (including no qualifications) (2).

*Smear history*: Target smear was first (1) *vs* Not the first/Don't know (2).

*Understanding*: Response (g) coded as 2; all other responses coded as 1.

*Final adjusted* *R*^2^=0.334;

* *P*<0.05;

** *P*=0.001;

*** *P*<0.001.

) in the group of women with inadequate smear test results. Ethnicity was not included in either of the regression analyses as there was too little variation in the sample to allow examination of this variable.

Higher state anxiety was predicted by greater perceived risk of developing cervical cancer (*β*=−0.154, *P*=0.042), and lower satisfaction with the information provided (*β*=−0.392, *P*<0.001) (see Table 3). Higher concern was predicted by perceiving the inadequate smear test result as uninformative of current cervical health status (*β*=0.171, *P*=0.010), and again less satisfaction with the information provided about the smear test result (*β*=−0.493, *P*<0.001) (see Table 4).

Of the 180 women receiving an inadequate smear test result, four moved out of the Sheffield area in the following 3 months. Of the remaining 176, 142 (81%) reattended for a repeat smear test within 3 months. Attendance was not associated with any demographic variables but was predicted by state anxiety assessed within 4 weeks of the initial test result: the women who did not attend for a repeat test had higher anxiety (*t*(155)=2.58, *P*=0.011) in the period after receiving their initial inadequate smear test result (mean=43.2, s.d.=15.8) than the women who did attend (mean=36.4, s.d.=12.2) (see Table 5

**Table 5:** Frequencies of women receiving an inadequate cervical smear test result who attended or did not attend for a repeat smear test within three months, and predictors of attendance (mean (s.d.), % (*n*))

	**Attenders (*n*=142)**	**Nonattenders (*n*=34)**	**Test statistics**	***P*-values**
Age; in years: Mean (s.d.)	40.3 (12.8)	36.6 (12.1)	*t*=1.528	0.128
Education: % (*n*)				
No qualifications	15 (22)	24 (8)	*χ*^2^=3.221	0.2
GCSE, GCE (A and O levels)	42 (59)	50 (17)	(df=2)	
Degree and higher education	42 (60)	26 (9)		
(Missing)	1 (1)	0		
				
Smear history: % (*n*)				
First smear	10 (14)	21 (7)	*χ*^2^=3.005	0.083
Not first smear	89 (127)	79 (27)	(df=1)	
Don't know	1 (1)	0		
				
State Anxiety: Mean (s.d.)	36.4 (12.2)	43.2 (15.8)	*t*=2.575	**0.011**
				
Concern: Mean (s.d.)	8.6 (2.8)	8.9 (3.2)	*t*=0.631	0.529
				
Perceived stressfulness about next smear test: Mean (s.d.)	3.4 (1.9)	4.1 (2.2)	*t*=1.796	0.074
				
Satisfaction with information: 4-item scale: Mean (s.d.)	19.7 (5.3)	17.8 (6.7)	*t*=1.787	0.076
				
Perceived risk of developing cervical cancer: % (*n*)^a^				
(f) Much higher than average	2 (3)			
(g) A bit higher than average	15 (22)	15 (5)		
(h) Same as average	68 (96)	68 (23)	*Z*=0.899	0.369
(i) A bit lower than average	8 (12)	9 (3)		
(j) Much lower than average	4 (5)	9 (3)		
(Missing)	3 (4)			

a Mann–Whitney ‘*U*’-test.

*P*-values reaching statistical significance (*P*<0.05) in bold.

).

## DISCUSSION

Informing women that they have an inadequate smear test result is associated, at least in the short term, with raised levels of state anxiety and concern about the test result that are similar to the levels reported in women with borderline or mildly dyskaryotic smear test results (Maissi *et al*, 2004). Anxiety levels were higher the more women perceived themselves to be at risk for cervical cancer and the less satisfied they were with the information they had received about their test results. Concern about test results was predicted by perceiving the results as uninformative and feeling less satisfied with the information provided. Nonattendance within 3 months for a repeat cervical smear test was predicted by higher levels of state anxiety within 4 weeks of an initial inadequate smear test result.

Pragmatically, one of the most effective ways of reducing the amount of anxiety and concern associated with receipt of an inadequate smear test result is to reduce the number of smears classified as inadequate. It seems likely that this will be achieved by the use of liquid-based cytology (LBC), which in a recent study was shown to reduce the rates of inadequate smear test results from 9.1% at baseline, using conventional cytology, to 1.6% (Moss *et al*, 2004). Thus, while in the future the rates of inadequate smear tests results are likely to be far lower with LBC, it remains important to consider how to reduce the anxiety and concern that will continue to be experienced upon receipt of an inadequate smear test result, albeit in a smaller proportion of women than is currently the case.

The majority of women receiving a first or nonconsecutive inadequate smear test result were aware that the result was uninformative about their cervical health. This correct perception was nevertheless associated with a high level of concern and perceived risk of developing cervical cancer. This counter-intuitive finding may be explained in terms of a mismatch between the information that women are expecting to receive from cervical screening, and this particular test result (Cioffi, 1994; Renner, 2004). Women may initially see attendance for cervical screening as a means to prevent cervical cancer. ‘Peace of mind’ is a reason often given for attending screening programmes (Saidi *et al*, 1998). In common with people undergoing other screening procedures, women undergoing cervical screening are interested in monitoring the extent to which disease is present, and expect to be told either that there is no detectable disease, or that there is some evidence of disease (Nerenz *et al*, 1982). Those receiving an inadequate smear test result are instead given a result that is not framed in terms of whether disease is present or absent: their expectations are not met. This result does not provide the sort of information that those undergoing cervical, or indeed other types of screening, are seeking. Receiving an inadequate smear results may change women's expectations of the screening programme: receiving such a result may lead women to view screening more as a means to detect disease. The lack of match between the sort of information women seek from cervical screening, and that they are given following an inadequate test result may explain why women made anxious are less likely to reattend for a further smear test.

Satisfaction with information was the strongest predictor of both anxiety and concern about the test result, accounting for 14 and 21%, respectively, of unique variance. In-depth interviews with women receiving inadequate smear test results would be a useful starting point to ascertain what information they want. The results of the present study suggest that telling women not to worry is insufficient. Women were not told what their test result meant in terms of their absolute risks of having or developing cervical cancer. In part, this reflects the poor evidence base. A recent study suggested that women with multiple inadequate smear test results may have a slightly increased risk of developing high-grade CIN over a period of 5 years in comparison to other women with adequate smear test results (Hock *et al*, 2003). While the precise risks await more and better powered studies, there is a consensus that the risks of cervical cancer attributable to inadequate smear tests results are less than those attributable to borderline or mildly dyskaryotic smear tests results.

Relative risk information often has a disproportionately large emotional and behavioural impact, in the absence of absolute risk information (Gigerenzer, 2002). The challenge is therefore to communicate to women that while an inadequate smear test result might indicate a slightly elevated risk of cervical cancer, their absolute risks of developing this disease are still very small. Perceptions of the prevalence of a health threat affect how seriously it is perceived, with threats perceived as more common being seen as less serious (Jemmott *et al*, 1986). While women were informed that an inadequate smear test result was ‘quite common’, we do not know how they interpreted this statement. Informing women of the actual frequency with which this test result occurs may thus reduce some of the concern and anxiety it generates. Given that high state anxiety in those women receiving an inadequate smear test result predicted nonattendance for a repeat smear test, improvements in communicating these ideas should not only reduce the distress experienced by women receiving this result, but should also achieve higher reattendance and hence better clinical outcomes from the screening programme. Studies are now needed comparing different ways of presenting the results of inadequate smear test results to determine those that are most effective at providing appropriate reassurance and avoiding high levels of anxiety.

With regards to understanding the meaning of normal smear test results, almost a quarter of women receiving a normal test result erroneously reported that their test result meant they definitely did not have cervical cancer. More research is needed to evaluate different ways of presenting normal smear test results to avoid such false reassurance (Marteau *et al*, 2001).

### Limitations of the study

Given that the study was observational in design, it is not possible to infer whether the associations observed are causally linked and if so, the direction of the association. Thus for example, while it is plausible that perceiving a higher risk of cancer could result in more anxiety, it is also possible that raised anxiety may result in more threatening thoughts (Bower, 1981), including an increased perception of the risk of cervical cancer. Prospective studies, assessing baseline mood and perceptions, as well as experimental studies targeted at altering perceptions of risk will help to ascertain the causal nature of the observed associations between mood and cognition.

The generalisability of the results is limited by the sample. While the response rate was very good for a postal survey, women from ethnic minorities were under-represented in our sample. The present study had insufficient power to assess the possible moderating effects of ethnicity on psychological responses to HPV testing.

### Concluding comment

Receiving an inadequate smear test result raises state anxiety and concern, similar to levels seen in women with borderline/mildly dyskaryotic smear test results. Anxious women are less likely to attend for a repeat smear test within the recommended time frame. Given the huge numbers of women worldwide receiving such a test result each year, it must now be a matter of some urgency to develop ways of presenting such test results to avoid or reduce the anxiety and concern that they are now known to cause.
